# Primary Chest Wall Hydatid Disease: A Case Report with Multimodality Imaging Findings

**DOI:** 10.1155/2023/5313067

**Published:** 2023-04-13

**Authors:** Waleed Althobaity, Ayman Aldeheshi, Mnahi Bin Saeedan

**Affiliations:** ^1^Department of Radiology, King Faisal Specialist Hospital & Research Center, Riyadh, Saudi Arabia; ^2^Department of Pathology & Laboratory Medicine, King Faisal Specialist Hospital & Research Center, Riyadh, Saudi Arabia

## Abstract

Primary chest wall hydatid cyst is a very rare disease in endemic areas. This case report describes a 22-year-old male patient with a 3-year history of chronic left chest pain. He had a history of close animal contact in childhood. Chest computed tomography (CT) scan showed a left upper posterior paravertebral cystic mass with peripheral and intrinsic calcifications. Fluorine-18 fluorodeoxyglucose (F-18 FDG) positron emission tomography (PET) scan showed no significant FDG uptake. Magnetic resonance imaging (MRI) showed a left paravertebral cystic mass with daughter cysts and a peripheral low T2 wall, compatible with hydatid disease. Medical treatment was started, and a follow-up MRI showed rupture of hydatid cysts. The patient underwent surgical resection, and a hydatid disease diagnosis was confirmed by histopathologic examination. During the postoperative hospital course, the patient developed pneumothorax which was successfully treated with a surgical procedure. The patient was discharged with medical treatment (albendazole). In conclusion, this case highlights the importance of considering hydatid disease in the differential diagnosis of chest wall cystic masses, especially in endemic regions, and the value of multimodality imaging in diagnosis and treatment planning.

## 1. Introduction

Hydatid disease is caused by tapeworm parasites and is prevalent in sheep-rearing regions, such as the Middle East, Mediterranean regions, Africa, South America, and Australia. The most common causative organism of human hydatid disease is *Echinococcus granulosus* [1–3]. The liver is the most commonly affected organ, followed by the lungs. Hydatid cysts can occur in any organ and anatomical site of the human body, with or without liver and lung involvement. Hydatid cysts can cause symptoms from mass effects, rupture, or superinfection [1, 2, 4]. The rupture of hydatid cysts can lead to life-threatening anaphylactic reactions from the antigenic properties of the cyst fluid [3–5]. Thoracic extrapulmonary hydatid disease is uncommon and may involve the mediastinum, pleural space, chest wall, cardiovascular structures, or diaphragm [1, 2, 6]. We report multimodality imaging findings of a young male patient diagnosed with primary chest wall hydatid disease who presented with left chronic pleuritic chest pain and left paravertebral cystic mass.

## 2. Case Presentation

**Figure 1:**
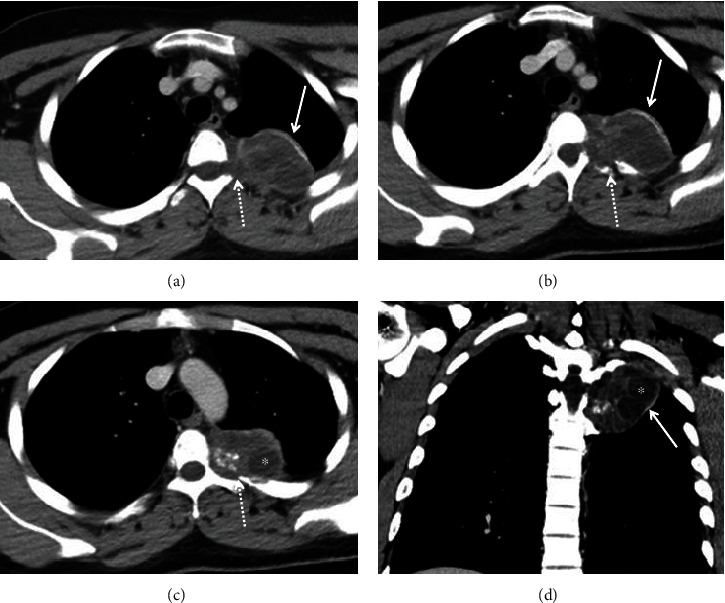
Axial (a–c) and coronal (d) images of contrast-enhanced CT exam show a left upper paravertebral cystic mass (arrows) with daughter cysts (asterisks), peripheral and intrinsic calcifications, adjacent ribs remodeling and erosions, and extension into adjacent left neural foramina (dashed arrows).

**Figure 2:**
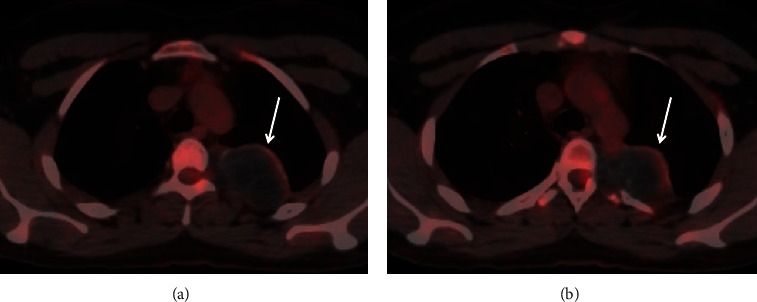
Axial fused PET/CT images (a, b) show a well-define left paravertebral cystic mass and heterogeneous cystic mass with no significant FDG uptake.

A 22-year-old male patient, with a history of close contact with goats and camels during childhood, presented with chronic left pleuritic chest pain for three years with no associated symptoms. The patient has had no significant medical or family history. His physical examination was normal. A computed tomography (CT) scan of the chest with intravenous (IV) contrast revealed a 4 × 6 × 6 cm cystic mass in the left thoracic paravertebral region extending from T3 to T5 vertebral bodies. The cystic mass had peripheral and intrinsic calcifications, cysts of different sizes and showed remodeling and erosion of adjacent ribs, with extension into the left T3-T4 and T4-T5 neural foramina (Figure 1). Fluorine-18 fluorodeoxyglucose positron emission tomography/computed tomography (F 18-FDG PET*/*CT) showed no significant FDG uptake in the lesion (Figure 2). The primary differential diagnosis was a nerve sheath tumor, and a magnetic resonance imaging (MRI) with IV contrast was recommended for further characterization.

**Figure 3:**
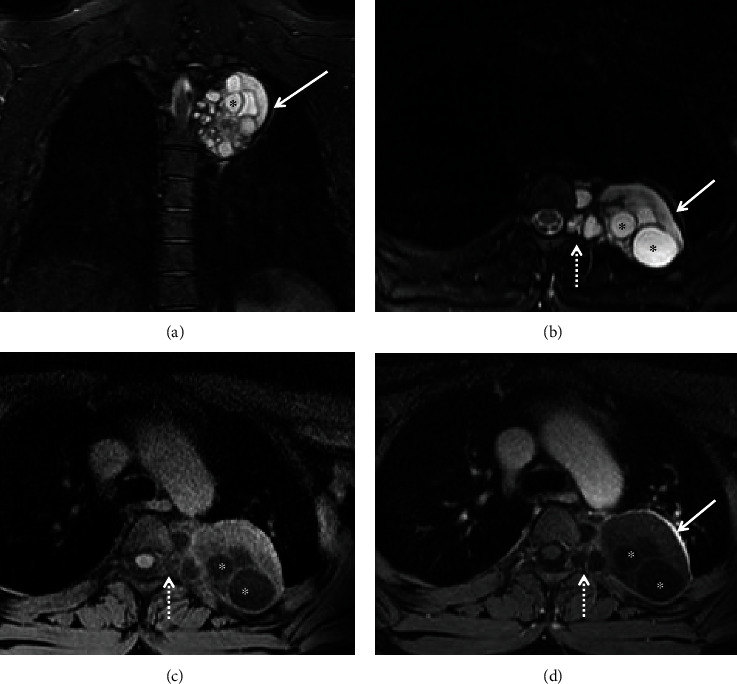
Coronal (a) and axial (b) T2-weighted images and axial T1-weighted images pre- (c) and post-IV contrast injection (d) show a left paravertebral hydatid cyst. The cystic mass shows numerous and variable in size daughter cysts (asterisks), the rim of low signal intensity on T2-weight images with enhancement in the postcontrast image (arrows), and extension into the adjacent neural foramina (dashed arrows).

The MRI showed numerous and varying sizes of intralesional daughter cysts with low T1 signal intensity and high T2 signal intensity compared to the large mother cyst matrix, which had intermediate T1 signal intensity and intermediate to high T2 signal intensity. The lesion also had a smooth, peripheral rim of low T2 signal intensity with postcontrast enhancement (Figure 3). There was no evidence of mural nodules or suspicious solid components, and no imaging signs of hepatic or pulmonary hydatid disease were present.

**Figure 4:**
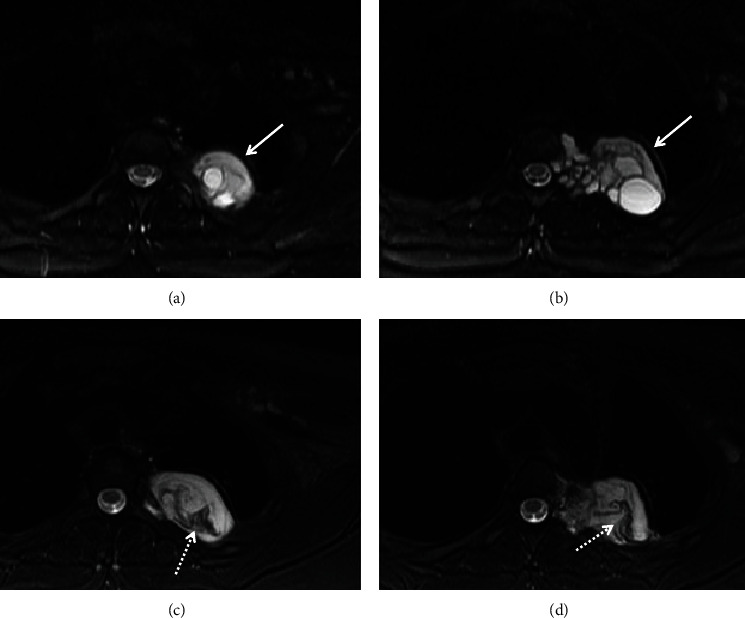
Axial T2-weighted images before medical treatment (a, b) show the left paravertebral hydatid cyst with numerous daughter cysts (arrows). Axial T2-weighted images postmedical treatment (c, d) show interval rupture of the daughter cysts and visualization of floating membranes (dashed arrows); these findings are compatible with contained rupture.

The imaging finding, specifically the classic appearance of daughter cysts on the MRI, strongly suggested a hydatid cyst. The patient's complete blood count and liver function test were normal. No hydatid serology or skin tests were requested. Oral albendazole was prescribed at a dose of 400 mg twice daily. Five months after starting medical treatment, a follow-up MRI showed the interval rupture of the daughter cysts and the development of low-signal floating membranes (Figure 4). A video-assisted thoracoscopic surgery was performed through two surgical ports to remove the cyst. Adhesions between the lung and the cyst were dissected. There was some injury to the lung pleura during the procedure. The cyst was isolated, soaked with hypertonic saline 7%, and aspirated after injection of hypertonic saline 7%. Cyst evacuation and cystectomy were performed and sent for histopathology. A 28 French chest tube was placed through the 7th intercostal space port and removed a few days after the surgery. The patient had a postoperative air leak, leading to an enlarging pneumothorax. A thoracoscopic assessment revealed a small air leak in the major fissure between the lateral aspect of the posterior apical segment and the lateral part of the lower lobe superior segment, which was located at the site of previous adhesiolysis. This was managed by performing a wedge resection of both the identified area and a mechanical pleurodesis with scratch. A chest tube was then inserted into the 5th intercostal space and positioned in the apical region. Serial postoperative chest radiographs showed a reduction in the size of the pneumothorax over time, with a small residual apical pneumothorax. The chest tube was clamped for four hours, and there was no change in the pneumothorax or air leak with coughing. The chest tube was then removed, and the patient was discharged. The patient was given albendazole for 9 months and is planning to continue the medication until follow-up imaging exams indicate no residual disease.

**Figure 5:**
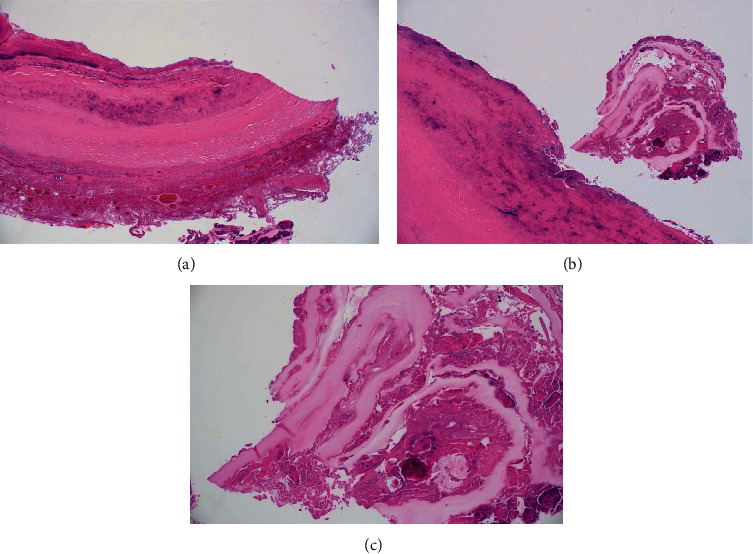
Histopathology images using hematoxylin and eosin (H&E) stains. Low power view (H&E 20x, (a)) shows the thick fibrous capsule with a mixed inflammatory infiltrate and granulation tissue. Medium power view (H&E 40x, (b)) shows the inner side of the capsule with a laminated cyst wall lining. A higher power view (H&E 200x, (c)) shows the laminated membrane structure. In the cyst wall, some areas demonstrated acellular laminated membranous structures with cellular debris and calcifications (b, c). The adjacent lung tissue was congested and showed a chronic inflammatory reaction. There were no viable protoscolices in the cyst wall and adjacent lung.

The histologic examination showed a thick fibrous capsule with a mixed inflammatory infiltrate and granulation tissue and acellular laminated membranous structures with cellular debris and calcifications in the cyst wall. The histologic findings are consistent with hydatid disease, and there was no evidence of viable protoscolices (Figure 5). A follow-up CT scan performed 3 months after discharge revealed a small, loculated left hydropneumothorax and no evidence of a residual hydatid cyst. At the last follow-up visit, 5 months after discharge, there were no active complaints, and the surgical wound was clean.

## 3. Discussion

Extrapulmonary thoracic hydatid disease is uncommon and can involve the mediastinum, pleural space, chest wall, cardiovascular structures, and diaphragm [1, 2, 6]. Primary chest wall hydatid disease is extremely rare and can affect bones, soft tissues, or both.

Previous cases of chest wall hydatid disease reported chest pain or chest wall swelling and symptoms lasting from a few months to several years. The chest wall hydatid disease diagnosis was often made postoperatively due to inconclusive or lack of preoperative imaging [7–11].

The MRI played a crucial role in diagnosing this case of hydatid disease by showing the classic appearance of daughter cysts. This triggered further questioning and revealed a history of close contact with animals. The MRI also documented the interval development of the contained rupture of the daughter cysts and the formation of a floating membrane (water lily sign) in response to medical treatment [2]. This case highlights the value of MRI in characterizing indeterminate thoracic cystic lesions and its potential impact on treatment decisions.

The life cycle of *Echinococcus granulosus* requires a definitive host, most commonly dogs, and an intermediate host, usually sheep. The parasite eggs are excreted in the host's feces, and humans can become accidental hosts by ingesting contaminated food or water. The larvae are released from the ingested eggs and are absorbed by the intestinal mucosa, commonly spreading to the liver through the mesenteric and portal veins. In around 15% of cases, the larvae bypass the liver filtration and gain access to the lungs [1, 3]. Hematogenous spread of *Echinococcus granulosus* to any part of the body can also occur with or without liver involvement [1, 3, 6, 12].

The hydatid cyst consists of three layers: the outer pericyst, the middle ectocyst, and the inner endocyst. The host's reactive inflammatory response forms the outer pericyst, which is a dense fibrous protective layer around the parasite. The ectocyst is an acellular, elastic laminated membrane in the middle. The endocyst is the innermost layer, made up of a single germinal epithelial layer, which houses daughter cysts within the larger mother cyst [1, 3].

Imaging plays a crucial role in detecting and diagnosing hydatid disease, particularly in cases where there is a history of exposure or immigration from an endemic area [1–3]. Hydatid cysts are typically classified into four types based on their imaging appearance [2]: type I: simple hydatid cysts appear as homogeneous, fluid-filled lesions with homogeneous low signal on T1-weighted MRI images and high signal on T2-weighted MRI images with a dark rim on MRI. On CT scans, they show homogeneous fluid attenuation. Type II: daughter cysts are smaller cysts within the larger mother cyst and are usually located at the periphery. They tend to have lower attenuation on CT scans compared to the mother cyst matrix. The shape of daughter cysts can be irregular, and they can occupy a large portion of the mother cyst. Type III: hydatid cysts with near-complete calcification indicate the death of the cyst. Type IV: hydatid cysts are complicated by rupture and/or superinfection. The causes of cyst rupture can include cyst degeneration, response to treatment, and trauma. Hydatid cyst rupture can be contained or can communicate with surrounding structures. Intracystic floating membranes, manifesting as serpentine linear structures of low attenuation on CT and low signal intensity on MRI, are indicative of contained rupture. This imaging sign “water lily sign” is highly suggestive of a hydatid cyst [2].

Casoni's intradermal skin test and indirect haemagglutination test (IHA) can detect the presence of antibodies to the parasite, but they have varying sensitivity (57–93%) and limited specificity. The IHA serology test has a reported sensitivity range of 66-100% and can be used as a screening test [13]. No hemagglutination tests were performed for the presented case, as the provisional diagnosis was made based on patient history and radiologic investigations. Additionally, hydatid serology is only valuable when it is positive, and a negative serologic test does not exclude the diagnosis.

Percutaneous transthoracic aspiration should be avoided or done with caution as it may cause allergic reactions or spillage of cyst content. Surgical resection is the preferred treatment for most patients with hydatid disease, while medical therapy with albendazole or mebendazole is used in addition to surgery. Medical therapy is the primary option for patients who cannot tolerate surgery or those with recurring hydatid disease or small cysts [14, 15].

Prolonged air leak, wound infection, empyema, seroma, and fistula formation are some of the most commonly observed postsurgical complications following chest hydatid cyst removal. Among all complications, the air leak with residual space and wound infection had the highest frequency, as was seen in our case [16].

Antiparasitic agents are also used as a supplement to surgical treatment. Albendazole, mebendazole, and praziquantel are antiparasitic agents that can be used. We began the albendazole regimen for one standard cycle immediately after surgery [14, 15].

## 4. Conclusion

Primary chest hydatid disease is a rare occurrence. We reported a case of primary chest hydatid disease in a young male patient with a history of close contact with goats and camels, who presented with chronic left pleuritic chest pain and a left paravertebral cystic mass showing typical MRI features of a hydatid cyst. The presence of intracystic daughter cysts and floating membranes (water lily sign) on imaging are typical features of hydatid disease. This rare case highlights the importance of including hydatid disease in the differential diagnosis of thoracic cystic masses and the utility of chest MRI, particularly in patients who have a history of exposure to endemic areas or close contact with intermediate hosts.

## Data Availability

No underlying data was collected or produced in this study.
